# Cerebellar cognitive affective syndrome in patients with spinocerebellar ataxia type 10

**DOI:** 10.1371/journal.pone.0319505

**Published:** 2025-03-03

**Authors:** Angel Omar Romero-Molina, Gabriel Ramirez-Garcia, Amanda Chirino-Perez, Gustavo Padron-Rivera, Carlos Roberto Hernandez-Castillo, Maria Guadalupe Garcia-Gomar, Diana Laura Torres-Vences, Juan Fernandez-Ruiz

**Affiliations:** 1 Instituto de Neuroetologia, Universidad Veracruzana, Xalapa, Veracruz, Mexico; 2 Laboratorio de Neuropsicologia, Facultad de Medicina, Universidad Nacional Autonoma de Mexico, Ciudad de Mexico, Mexico; 3 Faculty of Computer Science, Dalhousie University, Halifax, Canada; 4 Escuela Nacional de Estudios Superiores Unidad Juriquilla, Universidad Nacional Autonoma de Mexico, Juriquilla, Queretaro, Mexico; Isfahan University of Medical Sciences, IRAN, ISLAMIC REPUBLIC OF

## Abstract

**Background:**

Spinocerebellar ataxia type 10 (SCA10) is an autosomal dominant cerebellar ataxia, characterized by epilepsy, ataxic symptoms, and cognitive impairments linked to Cerebellar Cognitive Affective Syndrome (CCAS). The Cerebellar Cognitive Affective Syndrome Scale (CCAS-S) has been developed to identify CCAS across various cerebellar pathologies.

**Objective:**

To determine whether patients with SCA10 exhibit CCAS using the CCAS-S, and to compare its effectiveness with the Montreal Cognitive Assessment (MoCA). A secondary objective was to evaluate the effect of demographic and clinical data on CCAS-S performance.

**Method:**

Fifteen patients with SCA10 and fifteen matched controls underwent assessments using the CCAS-S, the MoCA, the Scale for the Assessment and Rating of Ataxia (SARA), and the Center for Epidemiologic Studies Depression Scale (CES-D). Diagnostic accuracy was analyzed using ROC curve analysis, comparing total and subcategory scores between groups. Demographic and clinical data were examined for relations with CCAS-S scores.

**Results:**

The CCAS-S effectively distinguished cognitive impairments in SCA10 patients, showing satisfactory sensitivity and specificity (AUC of 0.83). Although no significant differences were found in the AUCs between CCAS-S and MoCA (p =  0.45), the CCAS-S demonstrated a significantly larger effect size in the comparison between patients and control group (d =  2.33). Cognitive performance was poorer in patients than in controls (p =  < 0.001), with depressive symptoms and age having a significant impact on CCAS-S outcomes.

**Conclusions:**

Patients with the SCA10 mutation exhibit CCAS. Besides the significant cognitive impairment, also detected by MoCA, the CCAS-S score was significantly affected by indicators of depressive mood and age, highlighting the importance of considering these variables during outcome analyses.

## Introduction

Spinocerebellar ataxia type 10 (SCA10) is one of the most prevalent forms of spinocerebellar ataxia (SCA) in the Mexican population [1,2]. This condition is caused by a mutation in the ATXN10 gene, characterized by an expansion of ATTCT pentanucleotide repeats in intron 9 [3]. Clinical manifestations encompass epilepsy and slowly progressive ataxic symptoms, including gait disturbance, imbalance, loss of coordination, and dysarthria [4], which can be explained by the extensive degeneration of the cerebellum in these patients [5].

Previously, the function of the cerebellum was thought to be limited to motor control. However, clinical and imaging studies have identified its involvement in various cognitive and affective processes [6–8]. Consequently, the concept of cerebellar cognitive affective syndrome (CCAS) has emerged [9–11], characterized by deficits in executive functions, visuospatial functions, linguistic processing, and affective regulation [12,13]. The Cerebellar Cognitive Affective Syndrome Scale (CCAS-S) was developed as a screening test designed to assess ten cognitive domains in patients with cerebellar disorders [14]. Although the scale was initially tested in the US, it has since been validated in several other countries [15], including Spanish-speaking ones [16]. Owing to its sensitivity, the CCAS-S has been widely employed for detecting CCAS across various pathologies associated with cerebellar impairment [17], including different types of SCA (types 1, 2, 3, 6, 7) [18–21]. Despite SCA10 being a type frequently found in countries like Mexico and Brazil [22], previous studies on cognitive deficits in patients with SCA10 have not characterized the CCAS-S in these patients [23].

In this regard, the objectives of the present study were: (i) to characterize CCAS in patients with SCA10 using the CCAS-S and compare its clinical utility with the widely used Montreal Cognitive Assessment (MoCA). Our evaluation showed that patients with SCA10 had poorer cognitive performance in CCAS-S than control subjects. Furthermore, the CCAS-S proved to be an adequate and easily administered screening test that facilitates the characterization of CCAS in patients with SCA10. (ii) To correlate the cognitive performance of patients with SCA10 with their demographic and clinical characteristics. These analyses showed that age at examination and indicators of depressive mood may impact the CCAS-S total score.

## Materials and methods

### Participants

This study included 15 patients (9 women, mean age 51.06 years ±  11.39 SD). All patients had a proven pentanucleotide (ATTCT) repeat within the expanded range. Five patients reported the presence of seizures (confirmed by relatives). Two mutation carriers (younger than 60 years) had SARA scores of one, and one mutation carrier (older than 60 years) had SARA score of four, therefore were considered as asymptomatic [24]. Fifteen control subjects were matched for age, gender, and education level (9 women, mean age of 52.2 years ±  12.29 SD) to the patient sample (Table 1). All participants had no history of neurological or psychiatric diseases. This study was approved by the Research Committee of the Facultad de Medicina, Universidad Nacional Autónoma de México (approval number 007/2014). Informed consent was obtained in writing from all participants. The study adhered to the ethical guidelines of the Helsinki Declaration for human research. The recruitment period for this study was from March 1, 2022, to November 7, 2023.

**Table 1:** Demographic and clinical data of patients and control group.

	SCA10 patients (n = 15)	Control group (n = 15)	p_value_
**Age at examination (years), mean (± SD)**	51.06 (± 11.39)	52.2 (± 12.29)	0.79
**Education, mean (± SD)**	9.86 (± 2.87)	9.93 (± 2.78)	0.94
**Age at onset (years, n = 12), mean (± SD)**	37.5 (± 8.17)	–	
**Disease duration (years)**	10.34 (± 10.04)	–	
**SARA, mean (± SD)**	12.1 (± 7.35)	–	
**MoCA, mean (± SD)**	21.46 (± 4.98)	26.06 (± 3.55)	0.0002
**Depressed mood (CES-D), mean (± SD)** **Individuals under cutoff point, n (%)**	10.06 (± 7.43) 3 (10%)	9.06(± 4.92) 1 (3.3%)	0.68

Demographic and clinical data of patients and the control group. For clinical variables, we present the total raw score. SARA =  Scale for the Assessment and Rating of Ataxia, MoCA =  Montreal Cognitive Assessment Test, CES-D =  Center for Epidemiologic Studies Depression Scale. Only for CES-D, we present the frequency and percentage of cases that rates under the cutoff point.

### Behavioral assessments

#### Clinical assessment.

To assess ataxic motor symptoms, the Scale for the Assessment and Rating of Ataxia (SARA) was used. SARA is a clinical scale that evaluates a range of different impairments in cerebellar ataxia. It consists of eight categories with cumulative scoring ranging from 0 (no ataxia) to 40 (most severe ataxia) [25]. Additionally, to assess mood features, the Center for Epidemiologic Studies Depression Scale (CES-D) was used as an indicator of depressed mood [26].

#### Cognitive assessment.

The Cerebellar Cognitive Affective/Schmahmann Syndrome Scale (CCAS-S) [14] is a 10-item scale developed as a screening tool for Cerebellar Cognitive Affective Syndrome (CCAS) in individuals with cerebellar disease or injury. The CCAS-S assesses various cognitive domains, including semantic fluency, phonemic fluency, category switching, verbal memory, digit span forward and backward, cube drawing, similarities, the Go/No-Go test, and affect. Each item yields a raw score, and the total score can range from 0 to 120, with higher scores indicating better performance. Performance on each test is scored as pass or fail based on item-specific thresholds, resulting in a total score out of 10, where higher scores indicate a greater number of failures. The number of failed tests determines the diagnosis: zero failed tests indicate the absence of CCAS, one failed test indicates possible CCAS, two failed tests indicate probable CCAS, and three or more failed tests indicate definite CCAS. Version 1A of the scale was implemented in this study, which has already been tested in the Mexican healthy population and patients with cerebellar lesions [17]. Additionally, the MoCA [27] was administered as a comparative cognitive screening measure, commonly used in clinical and research settings. This instrument evaluates several domains, including attention, executive functions, memory, language, visuoconstructive abilities, calculation, and orientation. The maximum score achievable on this test is 30. All participants were evaluated with the CCAS-S [14] and MoCA. Finally, to assess articulation speed, participants were asked to repeat the word “PATA” for 10 seconds [28]; two back-to-back assessments were made, and the mean score of both attempts was calculated.

#### Statistical analysis.

To explore and compare the screening accuracy of the CCAS-S and MoCA tests in patients, a ROC curve analysis was implemented, where the area under the curve was calculated for each assessment, and also curves were compared between both screening tests. ROC analyses were also performed for each task included in the CCAS-S.

The standardized residuals were calculated for all the variables, and to determine the normality distribution, the Shapiro-Wilk normality test was applied (p <  0.05). Depending on the normality of the data, either the t-test or the Mann-Whitney U-test was conducted for group comparison. Cognitive scores were corrected through linear regression using the variables of age, sex and education as covariates. Verbal fluency scores were compared among groups with a linear regression using PATA scores as a control covariate. To account for the challenge of multiple comparisons, p-values were corrected using the False Discovery Rate (FDR) method (q =  0.05). Additionally, effect sizes were computed using Cohen’s d or r, as appropriate.

Finally, demographic and clinical data were correlated with CCAS-S scores using a simple correlation analysis, obtaining a correlation matrix. Additionally, we implemented a multiple linear regression model for patients with SCA10, using demographic and clinical variables such as age, education, SARA score and CES-D score as predictors. These analyses were conducted using R version 4.0.5 and RStudio version 2021.09.0 Build 351.

## Results

### Indicators of diagnostic accuracy in the cognitive screening tests

**Fig 1:**
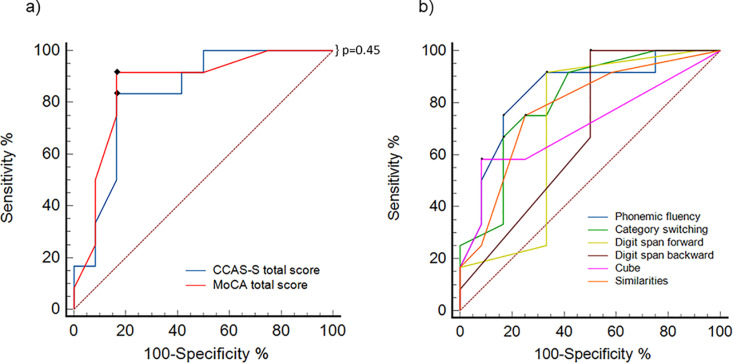
ROC curves analyses. (a) Comparison of ROC curves using MoCA and CCAS-S total scores. The black dots represent the identified optimal cut-off points, plotted in the sensitivity vs. specificity space. The X-axis (100-Specificity %) ranges from 0 to 100, where a value closer to 100 indicates lower specificity and a value closer to 0 indicates higher specificity. On the Y-axis (Sensitivity %), higher values correspond to greater sensitivity. Dotted red line represents an area under the curve of 0.5. b) ROC curves showing significant differences between the Control group and patients using each subtest of the CCAS-S (see Results for details).

Before analyzing the CCAS-S results comparison between patients and controls, we tested the sensitivity and specificity of the scale in patients with SCA10 to determine a cut-off point for their cognitive performance. Since three patients were diagnosed as asymptomatic and did not present ataxic symptoms during the SARA evaluation, they were excluded from the ROC curve analysis to maintain the integrity of the sensitivity and specificity characteristics of the ROC curve. The ROC analysis (n =  12) showed that CCAS-S (AUC (SE) =  0.83 (0.08), p =  0.0002) and MoCA (AUC (SE) =  0.86 (0.08), p =  < 0.0001). The comparison between the ROC curves of CCAS-S and MoCA did not show statistically significant differences (p =  0.45), which implied a similar test accuracy for both instruments. The maximum value of Youden’s index J (CCAS-S = 0.66; MoCA =  0.75) indicated the optimum cut-off point for CCAS-S at ≤  82 and for MoCA at ≤  25 (Fig 1a). For the CCAS-S, all subtests that had shown statistically significant differences between patients and controls were also taken into account to calculate the effectiveness indices: phonemic verbal fluency (AUC (SE) =  0.84 (0.08), p =  0.0001), category switching (AUC (SE) =  0.8 (0.09), p =  0.0007), digit span forward (AUC (SE) =  0.71 (0.11), p =  0.07), digit span backward (AUC (SE) =  0.68 (0.10), p = 0.07), cube drawing (AUC (SE) =  0.71 (0.09), p =  < 0.03), similarities (AUC (SE) =  0.77 (0.09), p =  < 0.004) (Fig 1b).

The ROC curve analysis revealed that the CCAS-S scores for three asymptomatic patients, without ataxic symptoms, were below the established cutoff. Therefore, they were included in subsequent analyses.

### Cognitive performance

We found that patients performed worse in both scales, the CCAS-S and the MoCA, compared to the control group. Similarly, patients had higher total fail scores in the CCAS-S than the control group. In the single-item CCAS-S analysis, we found that patients presented significant differences in the tests of phonemic verbal fluency, category switching, digit span forward, digit span backward, cube drawing, and similarities (Table 2).

**Table 2:** Cognitive scores of SCA10 patients and control participants.

Cognitive measure	SCA10 (n = 15)			Control group (n = 15)			Statistic	P_value_	P_FDR_	Effect size
	Mean±SD	Median (IQR)	Failed individuals n (%)	Mean±SD	Median (IQR)	Failed individuals n (%)				
MoCA total score	21.4 ± 4.9	23 (4.5)	13 (87%)	26.06 ± 3.5	26 (3.5)	4 (26%)	196	<0.001	<0.001	0.63
CCAS-S total score	77.8 ± 9.4	77 (6)	13 (87%)	95 ± 11.9	95 (15)	2 (13.3%)	6.40	<0.001	<0.001	2.33
CCAS-S total fails	5.6 ± 2.4	6 (2)	13 (87%)	2.5 ± 2.1	2 (2)	5 (33.3%)	−4.59	<0.001	<0.001	−1.67
**CCAS-S Cognitive domains**										
Semantic verbal fluency	20.3 ± 3.5	21 (5)	2 (13.3%)	21 ± 4.6	22 (7.5)	3 (20%)	130	0.48	NS	0.13
Phonemic verbal fluency	8.7 ± 3.1	8 (3.5)	10 (66.6%)	13.3 ± 3.1	13 (2.5)	2 (13.3%)	4.26	<0.001	0.001	1.55
Category Switching	9.06 ± 3.5	9 (3.5)	8 (53.3%)	12.4 ± 2.7	14 (2.5)	3 (20%)	3.30	0.002	0.005	1.20
Digit span forward	4.7 ± 0.7	5 (0)	14 (93.3%)	5.4 ± 0.9	6 (1.5)	6 (40%)	2.50	0.01	0.029	0.91
Digit span backward	3.2 ± 0.59	3 (1)	10 (66.6%)	4 ± 1.06	4 (2)	7 (46.6%)	2.55	0.01	0.029	0.93
Cube drawing	12 ± 2.4	11 (4.5)	8 (53.3%)	14.5 ± 1.3	15 (0)	1 (6.6%)	4.01	<0.001	0.001	1.46
Verbal memory	9.06 ± 3.5	9 (3)	11 (73.3%)	11.4 ± 3.15	13 (4.5)	5 (33.3%)	2.09	0.04	NS	0.76
Similarities	5.2 ± 1.5	5 (1.5)	12 (80%)	7.07 ± 1.26	7 (2)	5 (33.3%)	3.87	<0.001	0.001	1.41
Go/No-Go	1.13 ± 0.8	1 (1.5)	4 (26%)	1.46 ± 0.5	1 (1)	0 (0%)	1.61	0.11	NS	0.59
Affect	4.46 ± 1.06	5 (1.5)	6 (40%)	4.86 ± 1.06	5 (2)	5 (33.3%)	1.06	0.29	NS	0.38

Cognitive scores included MoCA total score, CCAS-S total score, CCAS-S total Fails score and the CCAS-S subtests between patients and controls. The label “failed individuals” indicates the number and percentage of participants who scored below the cut-off point in each test; ≤  25 for MoCA and ≤  82 for CCAS-S. In the CCAS-S total fails section, failed individuals are those participants who obtained 3 or more failures, resulting in a definitive CCAS classification. NS =  Not significant.

Furthermore, to account for potential dysarthria symptoms among patients, articulation speed was assessed using the PATA test. Patients (mean 25.26 ±  7.25 SD) exhibited significantly slower articulation rates (U =  198, p =  0.0001, r =  0.64) compared to the control group (mean 36.2 ±  5.27 SD), prompting adjustments in the verbal fluency tests based on PATA scores. Following these adjustments, no statistically significant differences were observed in the tasks that previously showed differences: phonemic verbal fluency (t =  1.89, p =  0.069, d =  0.69) and category switching (t =  1.98, p =  0.056, d =  0.72).

Since no statistically significant differences were found between the AUCs of the CCAS-S and MoCA, we decided to assess whether there were differences in the effect sizes of the comparisons between patients and controls for each of the two scales. It was found that both the CCAS-S and MoCA had a standard error (SE) of 0.37 and a difference in SE of 0.53. A comparison of effect sizes revealed statistically significant differences (z =  -3.19, p =  0.004), with the CCAS-S (d =  2.33) showing a larger effect size than the MoCA (r =  0.63).

**Fig 2:**
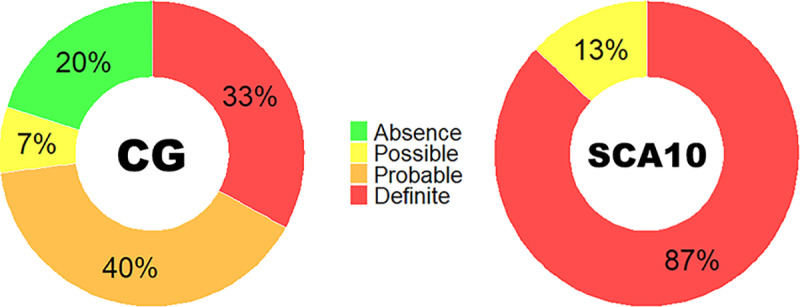
Percentage of CCAS-S diagnosis classification between groups. Percentage of participants in the control (CG) and patient (SCA10) groups with the characterization of “Definite,” “Probable,” “Possible,” and “Absence” of CCAS derived from the CCAS-S.

Finally, the proportion of SCA10 patients who presented a definitive positive CCAS was 87%, while 13% had a possible CCAS. On the other hand, for the control group, 33% had a definitive positive CCAS, 7% had a possible CCAS, 40% had a probable CCAS and 20% showed negative CCAS (Fig 2).

### Relation between CCAS and the clinical variables

Finally, a simple correlation analysis was conducted on clinical and demographic variables, enabling us to generate a correlation matrix of the variables and observe their associations. After FDR correction, the variables that showed statistically significant correlations were: CCAS-S and CES-D (Rho =  −0.76, p_FDR_ =  0.01); CCAS-S and age at examination (r =  −0.7, p_FDR_ =  0.03); and SARA and disease duration (Rho =  0.87, p_FDR_ <  0.001). The statistics and values of the correlation matrix are provided in full in a table in the supplementary material (S1a Fig and S1 Table).

Based on these findings, a multiple linear regression model was implemented to evaluate independent variables that could serve as predictors of the CCAS-S outcome. The variables selected for the model were age, education, disease duration, SARA score, and CES-D score. However, when testing the assumptions of this model, the SARA and disease duration variables exhibited collinearity and had the highest variance inflation factors (VIF). To determine which variable to remove from the model, we obtained the values of the Akaike Information Criterion (AIC) and the Bayesian Information Criterion (BIC). It was found that the model without the disease duration variable presented the lowest AIC and BIC values (model without SARA: AIC/BIC =  97.38/101.62, model without disease duration: AIC/BIC =  97.16/101.41). Thus satisfying all the assumptions of the multiple linear regression analysis, the chosen model was: CCAS-S ~  Age +  Education +  SARA +  CES-D. This model was statistically significant (R = .890, R² = .793, SE =  5.06, p =  0.002), with Age (Beta =  −0.39, t =  −2.27, p =  0.04), Education (Beta =  0.26, t =  1.46, p =  0.17), SARA (Beta =  −0.17, t =  −1.04, p =  0.32), and CES-D (Beta =  −0.40, t =  −2.34, p =  0.04). This indicates that age at examination and CES-D scores are significant predictors of CCAS-S performance (S1b Fig). These findings align with the variables that showed statistically significant correlations, suggesting that in this sample, age and CES-D scores may influence CCAS-S performance.

Similarly, a multiple linear regression analysis was performed to assess the influence of variables on the MoCA (MoCA ~  Age +  Education +  SARA +  CES-D) as a follow-up comparison in this scale. However, no variable was found to be significant in this model (R = .411, R² = .169, SE =  5.37, p =  0.7).

## Discussion

Here we wanted to test if patients with the SCA10 mutation exhibited CCAS using the CCAS-S, and to compare the scale’s clinical utility with that of MoCA. The CCAS-S demonstrated satisfactory sensitivity and specificity, with an AUC of 0.83. Although no statistically significant differences were found in the AUC between the two scales, the CCAS-S showed a significantly larger effect size compared to MoCA in the comparison between patients and controls. Our evaluation subsequently revealed that the CCAS-S identified worse cognitive performance in patients with SCA10 compared to controls. Finally, demographic and clinical variables were analyzed in relation to the overall CCAS-S score, revealing that age at examination and CES-D may impact the CCAS-S total score.

Following the description of CCAS, different studies have been conducted to describe this syndrome in various diseases involving the cerebellum [29–31], including SCA10 [4,23,32]. The implementation of this screening tool has allowed the identification of CCAS with high sensitivity and accuracy in other types of SCA [18]. In our study, we implemented ROC curve analysis to verify the effectiveness of the scale in identifying CCAS in SCA10, which showed high sensitivity and specificity. Although the curve did not show significant differences compared to the MoCA curve, it is important to mention that this aligns with a previous SCA2 study [16], which suggests that the CCAS-S is a valuable tool for assessing cerebellar cognition, complementing the MoCA, which has low sensitivity in tests of mental flexibility. In this context, we suggest that both screening tests might be complementary for better identification of CCAS. However, having confirmed that the effect size of the CCAS-S is statistically larger than that of the MoCA, we propose that the CCAS-S is a more viable tool for assessing cognition in the presence of cerebellar impairments in patients with SCA10. In fact, the CCAS-S is capable of classifying CCAS as Possible, Probable, and Definitive, giving an additional feature to this scale compared to MoCA. Although there was a higher proportion of patients with a Definitive classification, there were also cases of controls who scored with Possible, Probable, and Definitive categorization. This introduces variability that could impact the sensitivity and accuracy of such classification [15]. It is essential to conduct an analysis of how failures are obtained in each of the scale’s domains to ensure that the cut-off points for each task are appropriate for providing a correct pass-or-fail categorization. It is observed that participants are close enough to pass the cut-off point, but a single failure can significantly impact their diagnostic characterization. For example, in semantic fluency, a person who produces 14 words and another who produces only 2 would both be considered failures. Even though their raw scores differ, both would receive an additional point in the diagnostic characterization according to the scale.

In this regard, it has been found that cases where participants show a classification of 1 or 2 (possible and probable CCAS) often overlap with the performance of the controls [21]. In our case, 87% of the patients showed a definitive CCAS categorization. However, 7% of the controls were classified as possibly having CCAS, and 40% as probably having it. In this sense, the CCAS-S has been limited to diagnosing primarily severe cases that score with 3 or more failures to grant a definitive classification. This is important as it suggests that the most useful diagnostic categories are definitive vs. no presence of CCAS.

Another important point to highlight is the fact that the three asymptomatic patients scored below the established cutoff for the CCAS-S. In addition, two of these patients also scored below the MoCA cutoff. This finding is interesting because it suggests that the CCAS-S may be capable of detecting early cognitive changes, even before motor symptoms appear. Similar findings have already been reported in SCA3 [19].

The consistency in the classification of both the CCAS-S and the MoCA is noteworthy, as both achieved similar characterization of mild cognitive impairment in a comparable percentage of patients (MoCA: 26% and CCAS-S: 33%). This is supported by the lack of differences between the AUCs of the ROC curves. These results align with previous findings in cerebellar focal lesions measured in stroke patients [17], where similar cutoff points were obtained for the MoCA and CCAS-S. Additionally, the CCAS-S subdomains were largely similar, with impairments in phonemic memory, category switching, digit span forward and backward, cube drawing, and the similarities task. This is important as it contributes to the ongoing discussion of whether patients with complex pathologies like SCA10 exhibit different performance than patients with focal lesions, a topic that has been previously debated [14]. These results are specific to a single scale, however, and a more in-depth cognitive analysis with a comprehensive neuropsychological battery is recommended to compare patients with neurodegenerative diseases like SCA and those with focal lesions like stroke.

Additionally, in our study, we identified worse performance in the raw total and fail scores of the CCAS-S in patients with SCA10 compared to the control group. Similar results have been shown in other SCAs; for instance, patients with SCA2 had poorer total and item-based performance compared to a control group. Furthermore, it was shown that both the raw and failed CCAS-S total scores were associated with the SARA scores, education, and the MoCA [16]. In addition, another study conducted in SCA3, SCA6, and Friedreich’s ataxia patients revealed lesser performance in patients compared to controls. Moreover, in an intra-group analysis, the patients with SCA3 exhibited more deficits compared to patients with the other two types of ataxia [21]. A more extensive study evaluated the CCAS-S in different types of ataxias such as SCA1, SCA2, SCA3, SCA6, SCA7, and SCA8, as well as pre-symptomatic individuals with SCA1 and SCA3 [18]. This study showed worse performance among patients compared to controls. These findings demonstrate that patients with cerebellar ataxia, such as SCA10, exhibit CCAS as part of their constellation of symptoms. The CCAS-S, having proven to be a sensitive test, can effectively assess and identify cerebellar cognitive impairments in these patients [14].

Furthermore, in our study, the SCA10 patients showed worse performance in the tasks of phonemic verbal fluency, category switching, digit span forward, digit span backward, cube drawing, and similarities. This coincides with the main objective of the scale, which is to corroborate the presence of deficits in executive function, linguistic processing, and spatial cognition [14]. Additionally, this evidence also coincides with reports carried out in other SCAs where the main affected skills are those of verbal fluency [18,20]. This is a critical aspect, as dysarthria is a significant symptom in SCA [22], reflecting the degree of neurological impairment, particularly in fine motor control and coordination of the muscles involved in speech. This results in reduced speech rate and increased duration of pauses while speaking [33]. Therefore, we strongly recommend correcting verbal fluency scores through articulation speed tests, which will provide a clearer assessment of symptom severity [17]. Additionally, the population we evaluated mainly came from a rural environment with low levels of education. Therefore, it is important to demonstrate that these screening scales are useful in culturally diverse contexts compared to where they were originally developed. Thus, observing that similar cutoff points and results were obtained as proposed in other SCAs [15,16] confirms that the administration of CCAS-S is useful in determining CCAS in patients with SCA10 [14].

A highlight of our analysis is the comparison of the total CCAS-S scores with various demographic and clinical variables examined in other studies [16,18]. In our model, we included demographic and clinical variables like age, education, SARA score, and CES-D score, which are important diagnostic characteristics of SCA [22,32]. However CES-D score and Age were identified as the only predictive variables for the total CCAS-S score. This is relevant because age is a variable associated with cognitive scores on the CCAS in SCAs [18]. An important point is that CCAS-S does not adjust patients’ scores for age or educational level, which suggests the need to consider these variables in data analysis [21]. Also, a negative relation was found between the CCAS-S and the CES-D scale, which assesses depressive mood traits, indicating that the CCAS-S is linked to mood features. However, as discussed in other validation studies [15], evaluating the affective section of the CCAS-S is subjective and should be performed by a healthcare professional for optimal accuracy.

Finally, the study has some limitations. The small sample size limits the generalizability of the findings and the ability to detect subtle differences or analyze subgroups. The study’s cross-sectional design does not account for the progression of cognitive deficits over time, and further longitudinal studies are needed to assess whether the CCAS-S can detect early or progressive cognitive changes in SCA10 patients.

## Conclusion

In conclusion, patients with the SCA10 mutation exhibited CCAS. In addition to the significant cognitive impairment, which was also detected by the MoCA, the CCAS-S score was significantly affected by indicators of depressive mood. This highlights the distinctive cognitive and affective impairments observed in patients with cerebellar damage. In this sense, the CCAS-S is an adequate and easily administered screening test that facilitates the diagnosis of CCAS in patients with SCAs, including SCA10. Therefore, it can be considered an important option for determining CCAS when a rapid assessment of cognitive impairment is required. However, this assessment should be complemented with other clinical motor and mood scales, as well as a complete battery of neuropsychological tests, to reveal all motor and non-motor deficits that may be present in patients.

## Supporting information

S1 Fig Correlation matrix and multiple lineal regression analysis between CCAS-S and clinical and demographic variables.
a) Correlation matrix between demographic and clinical variables. Blue represents the threshold between positive correlations and red represents negative correlations. Asterisks indicate correlations that were statistically significant after FDR correction (p <  0.05). b) Shows the influence of age at examination and CES-D score on the total CCAS-S score. The graph shows the relationship between multiple independent variables and the dependent variable, allowing the model’s structure and the interactions between variables to be displayed. This helps to understand how the prediction surface behaves. The color bar indicates how the CCAS-S score varies according to the variables, with points shifting towards yellow indicating higher CCAS-S values.
(TIF)

S1 Table Statistical correlation analysis between CCAS-S and clinical and demographic variables.
Statistical correlation analysis table between demographic and clinical variables. Statistically significant correlations before FDR correction are highlighted in bold. Asterisks indicate those that remain significant after FDR correction.
(DOCX)
