# Supplementary figures and images for: Cerebellar cognitive affective syndrome in patients with spinocerebellar ataxia type 10
Source: PLoS One. 2025 Mar 3;20(3):e0319505. doi: 10.1371/journal.pone.0319505 (PMC11875346; doi:10.1371/journal.pone.0319505)

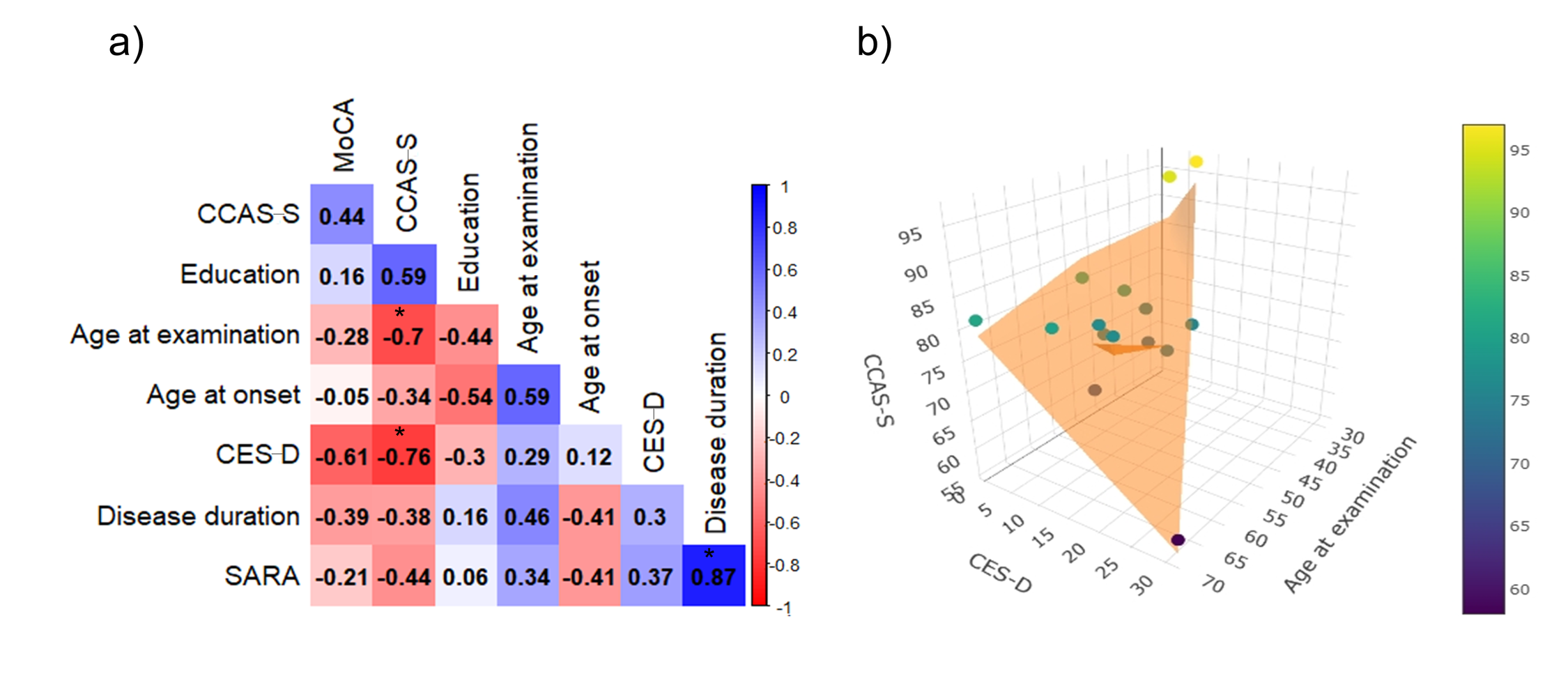

Supplement: S1 Fig — a) Correlation matrix between demographic and clinical variables. Blue represents the threshold between positive correlations and red represents negative correlations. Asterisks indicate correlations that were statistically significant after FDR correction (p < 0.05). b) Shows the influence of age at examination and CES-D score on the total CCAS-S score. The graph shows the relationship between multiple independent variables and the dependent variable, allowing the model’s structure and the interactions between variables to be displayed. This helps to understand how the prediction surface behaves. The color bar indicates how the CCAS-S score varies according to the variables, with points shifting towards yellow indicating higher CCAS-S values. (TIF) [file pone.0319505.s001.tif]
