# Supplementary material for: Cerebellar cognitive affective syndrome in patients with spinocerebellar ataxia type 10
Source: PLoS One. 2025 Mar 3;20(3):e0319505. doi: 10.1371/journal.pone.0319505 (PMC11875346; doi:10.1371/journal.pone.0319505)
Supplement: S1 Table — Statistical correlation analysis table between demographic and clinical variables. Statistically significant correlations before FDR correction are highlighted in bold. Asterisks indicate those that remain significant after FDR correction. (DOCX) [file pone.0319505.s002.docx]

**Table 1.** **Statistical correlation analysis.**

| Variables | | Coefficient | Statistic | P value | P_FDR_ value |
| --- | --- | --- | --- | --- | --- |
| CES-D | Age at examination | 0.29 | 397.90 | 0.29 | 0.38 |
| CES-D | Education | -0.3 | 728.76 | 0.27 | 0.38 |
| CES-D | Age at onset | 0.12 | 494.23 | 0.67 | 0.72 |
| CES-D | Disease duration | 0.3 | 390.57 | 0.27 | 0.38 |
| **CES-D** | **CCAS-S** | **-0.76** | **986.96** | **<0.001** | **0.01*** |
| **CES-D** | **MoCA** | **-0.61** | **900.80** | **0.01** | 0.09 |
| CES-D | SARA | 0.37 | 352.26 | 0.17 | 0.3 |
| Disease duration | Age at examination | 0.46 | 300.87 | 0.08 | 0.26 |
| Disease duration | Education | 0.16 | 469.29 | 0.56 | 0.63 |
| Disease duration | Age at onset | -0.41 | 786.82 | 0.13 | 0.28 |
| Disease duration | CCAS-S | -0.38 | 770.18 | 0.16 | 0.3 |
| Disease duration | MoCA | -0.39 | 780.44 | 0.14 | 0.29 |
| **Disease duration** | **SARA** | **0.87** | **74.52** | **<0.001** | **<0.001*** |
| **Age at examination** | **Age at onset** | **0.59** | **2.66** | **0.01** | 0.09 |
| **Age at examination** | **CCAS-S** | **-0.7** | **-3.49** | **0.003** | **0.03*** |
| Age at examination | Education | -0.44 | -1.74 | 0.1 | 0.26 |
| Age at examination | MoCA | -0.28 | -1.07 | 0.3 | 0.38 |
| Age at examination | SARA | 0.34 | 1.32 | 0.21 | 0.33 |
| Age at onset | CCAS-S | -0.34 | -1.29 | 0.21 | 0.33 |
| **Age at onset** | **Education** | **-0.54** | **-2.28** | **0.03** | 0.15 |
| Age at onset | MoCA | -0.048 | -0.17 | 0.86 | 0.86 |
| Age at onset | SARA | -0.41 | -1.63 | 0.12 | 0.28 |
| **CCAS-S** | **Education** | **0.59** | **2.65** | **0.01** | 0.09 |
| CCAS-S | MoCA | 0.44 | 1.74 | 0.10 | 0.26 |
| CCAS-S | SARA | -0.44 | -1.77 | 0.09 | 0.26 |
| Education | MoCA | 0.16 | 0.60 | 0.55 | 0.63 |
| Education | SARA | 0.055 | 0.19 | 0.84 | 0.86 |
| MoCA | SARA | -0.21 | -0.77 | 0.45 | 0.54 |

Statistical correlation analysis table between demographic and clinical variables. Statistically significant correlations before FDR correction are highlighted in bold. Asterisks indicate those that remain significant after FDR correction.
